# Regulations on palliative sedation: an international survey across eight European countries

**DOI:** 10.1093/eurpub/ckac153

**Published:** 2022-10-27

**Authors:** Eduardo Garralda, Csilla Busa, Éva Pozsgai, Veronika Osztromok-Lukacs, Agnes Csikós, Lukas Radbruch, Jeroen Hasselaar, Johan Menten, Sheila Payne, Claudio Adile, Flavia Hurducas, Carlos Centeno

**Affiliations:** ATLANTES Global Observatory of Palliative Care, Institute for Culture and Society, University of Navarra, Navarra, Spain; IdiSNA, Navarrese Centre for Sanitary Research, Pamplona, Spain; Department of Hospice-Palliative Care, Institute of Primary Health Care, University of Pecs Medical School, Pecs, Hungary; Department of Hospice-Palliative Care, Institute of Primary Health Care, University of Pecs Medical School, Pecs, Hungary; Department of Hospice-Palliative Care, Institute of Primary Health Care, University of Pecs Medical School, Pecs, Hungary; Department of Hospice-Palliative Care, Institute of Primary Health Care, University of Pecs Medical School, Pecs, Hungary; Department of Palliative Medicine, University Hospital Bonn, Bonn, Germany; Radboud University Medical Centre, Nijmegen, The Netherlands; Catholic University Leuven, Leuven, Belgium; International Observatory on End of Life Care, Lancaster University, Lancaster, UK; La Maddalena Cancer Center, Palermo, Italy; Hospice Casa Sperantei, Bucharest, Romania; ATLANTES Global Observatory of Palliative Care, Institute for Culture and Society, University of Navarra, Navarra, Spain; IdiSNA, Navarrese Centre for Sanitary Research, Pamplona, Spain

## Abstract

**Background:**

Palliative sedation is a commonly accepted medical practice. This study aims to clarify how palliative sedation is regulated in various countries and whether this may impact its practice.

**Methods:**

An online survey requesting regulations on palliative sedation was conducted in Belgium, Germany, Hungary, Italy, The Netherlands, Spain, Romania and the UK. Purposive sampling strategy was used to identify clinicians from different medical fields and legal experts for each country. Regulations were analyzed using the principles of the European Association for Palliative Care Framework on palliative sedation. Country reports describing how palliative sedation is regulated were elaborated.

**Results:**

One hundred and thirty-nine out of 223 (62%) participants identified 31 laws and other regulations affecting palliative sedation. In Spain, 12 regional laws recognize palliative sedation as a right of the patient at the end of life when there are refractory symptoms. In Italy, the law of informed consent and advance directives specifically recognizes the doctor can use deep sedation when there are refractory symptoms. There are also general medical laws that, while not explicitly referring to palliative sedation, regulate sedation-related principles: the obligation of doctors to honour advance directives, informed consent, the decision-making process and the obligation to document the whole process. In Germany, the Netherlands and the UK, palliative sedation is also regulated through professional guidelines that are binding as good practice with legal significance.

**Conclusions:**

Palliative sedation is considered in the general law of medical practice, in laws regarding the patient’s autonomy, and through professional guidelines.

## Introduction

Palliative sedation (PS) is a medical intervention aimed at relieving refractory suffering at the end of life through the monitored and proportional use of medications intended to reduce the patient’s consciousness.^1^ The European Association for Palliative Care (EAPC) acknowledges PS as an important intervention for patients experiencing refractory symptoms, and recommends that carefully developed procedural guidelines are needed for PS to be conducted safely and optimally.^2^ However, besides clinical guidelines, other factors such as their frequency of use of PS practice, professional attitudes or the indirect influence of regulations that restrict and condition certain practices may influence the commonalty and quality of PS.

The regulation of PS may facilitate the distinction between PS and euthanasia. Philosophically, PS is correctly interpreted as allowing death with permissible treatment of refractory symptoms (justified under the doctrine of double effect). The legal and conceptual distinction is relevant to countries that permit such practices (and to all) as clinicians may be reluctant to PS because they believe it is equivalent to active euthanasia. Other professionals may be concerned about a sanction for practicing PS or for misreporting their practice, depending on the country’s legal situation regarding euthanasia.^3^ Furthermore, there is assumed to be variability in the performance of PS across countries, and uncertainty associated with end-of-life circumstances, may add particular relevance to the way sedation is regulated.

According to the World Health Organization, regulation for health includes: (i) government rules accompanied by mechanisms for monitoring and enforcement (laws and legal tools); (ii) any form of direct state economic intervention (taxes, quotas or pricing mechanisms); or (iii) mechanisms of social control (carried out by non-government actors that do self-regulate, for instance, a health professional group to self-regulating rules of conduct for its members).^4^ Norms emanated from regulation may be non-binding for non-government sourced regulations, meaning they have legal significance, but are not mandatory. This last regulation may include the ‘doctrine’ (scientific evidence), sometimes materialized in professional guidelines, and the costume (daily practice) which still, in a broad sense, make up the regulation.

It is unknown whether PS falls under the remits of regular health care practice (or adheres to its principles such as the informed consent, obligation of doctors to honour advance directives), or, alternatively, whether it falls under specifically developed regulations, which may ameliorate practitioners’ fear of legal liability of PS. As part of an European project on PS, this study aims to identify the legal and professional regulations affecting PS across eight European countries: Belgium, Germany, Hungary, Italy, the Netherlands, Romania, Spain and the UK.

## Methods

### Survey design

An online search to find any questionnaires on the performance of PS was undertaken in PUBMED and Google Scholar, in English, for the period 2005–20. Search terms were: (‘palliative sedation’ AND ‘survey’). It provided 19 studies. A draft of six questions were discussed with the consortium members, reduced to four, and included in the final protocol. The survey was designed in Survey Monkey and requested if there was a legal framework for PS in the country, the type of regulation, the name and link, and the level of geographical application (Supplementary Appendix 1).

### Population, sample and recruitment

A purposive sampling strategy of key clinicians and other key persons was used by each consortium partner.

#### Key clinicians

Eighteen clinicians per country from a diverse range of groups who may perform PS: doctors and nurses, working in palliative care, anaesthesiology, intensive care, internal medicine, oncology and general practice (table 1). We used the following sources for identifying potential participants: (i) The National Palliative Care Medical and Nursing Associations, (ii) members of workforces on PS, guideline or related articles’ authors, (iii) National Anaesthesiology, Intensive Care, Primary Care, Oncology and Internal Medicine Societies and (iv) National Societies’ official scientific or linked journals.

**Table 1:** Matrix of 18 expert clinicians suggested to complete the survey for each country

Field	Setting	Profile (*n*)
Anesthesiology	Hospital	Doctor (1) + Nurse (1)
Intensive care	Hospital	Doctor (1) + Nurse (1)
Internal medicine	Hospital	Doctor (1) + Nurse (1)
Oncology	Hospital	Doctor (1) + Nurse (1)
Primary care	Primary	Doctor (1) + Nurse (1)
Palliative care	Hospital	Doctor (1) + Nurse (1)
Palliative care	Home	Doctor (1) + Nurse (1)
Palliative care	Nursing home	Doctor (1) + Nurse (1)
Palliative care	Hospice	Doctor (1) + Nurse (1)

#### Key persons

Two key persons: (i) an individual with legal knowledge and (ii) an individual with national-level knowledge on PS. The first expert could be the legal advisor of the Official Medical/nursing College or equivalent body, whereas the National Palliative Care Association designated the second.

Professional backgrounds, field of medicine, setting, post, nomination source and information suggesting the adequacy of nomination were evaluated, and justification for the absence of certain groups was requested.

#### Recruitment

A letter of introduction, consent and invitation was sent in December 2019 by consortium members. Upon acceptance, the survey was sent by 30 January, reminded 15 February and data collection closed on 15 March 2020.

### Data analysis

Data were exported to an excel spreadsheet. Reported regulations were assessed independently by E.G. and C.B. to determine whether they made explicit reference to PS. Attention was paid to whether regulations mentioned the terms ‘palliative sedation’ (or other similar wording such as terminal, profound or deep sedation), a significant description of sedation, the level of consensus by participants in the country. Components described in the EAPC Framework for PS were employed as reference items,^2^ providing a framework to understand which PS principles were regulated (table 2). Regulations reported by respondents not explicitly addressing PS, but describing PS-related EAPC principles, were considered relevant. A narrative report on each country was validated among consortium members.

**Table 2:** Principles covered by the EAPC Framework for palliative sedation

Refractory symptom including psychological suffering Description of the type of patients Informed consent/decision capacity Procedure requirements Nutrition and hydration Duty to respect the patient’s right Transparency of the decision-making process Collegial decision-making Information from the relatives Documentation of the entire decision-making process in medical files Principle of proportionality Obligation of doctors to honour advance directives

## Results

In total, 139/223 (62%) participants completed the questionnaire: 124 key clinicians and 15 key experts. Several key clinicians and at least one key expert participated per country. The number of participants and their disciplinary profiles varied between countries (participants mean 19, min–max: 9–37 per country; disciplinary profile mean 10.5/19, min–max: 4–18) (table 3).

**Table 3:** Profiles of the participants in the study

			Countries^a^								Profiles
Field	Setting	Profess	Neth	Bel	UK	Ger	Rom	Ita	Hun	Spa	*n* profiles/*n* countries
Anaesthesiology	Hospital	Doctor	1	1	–	1	1	3	1	1	7/8
	Hospital	Nurse	–	–	–	–	–	–	–	–	0/8
Intensive care	Hospital	Doctor	–	–	–	–	1	1	2	1	4/8
	Hospital	Nurse	–	–	–	–	–	1	–	1	2/8
Internal medicine	Hospital	Doctor	–	–	–	–	–	1	–	1	2/8
	Hospital	Nurse	1	–	–	–	–	1	1	1	4/8
Oncology	Hospital	Doctor	1	–	–	–	3	2	3	1	5/8
	Hospital	Nurse	–	–	–	–	–	1	1	1	3/8
Primary care	Primary	Doctor	1	1	–	1	1	1	1	2	7/8
	Primary	Nurse	–	1	–	–	–	–	–	1	2/8
Legal expert	Any	Expert	2	3	3	2	1	1	2	1	8/8
Palliative care	Hospital	Doctor	2	1	2	1	4	6	4	1	8/8
	Hospital	Nurse	6	2	–	–	1	–	1	1	5/8
	Home	Doctor	–	1	2	1	2	5	1	1	7/8
	Home	Nurse	2	1	–	1	1	1	1	1	7/8
	Residence	Doctor	2	1	–	1	–	–	–	1	4/8
	Residence	Nurse	–	–	–	–	–	–	–	1	1/8
	Hospice	Doctor	3	1	9	1	1	13	1	1	8/8
	Hospice	Nurse	–	–	–	–	1	1	–	1	3/8
Profiles	–	–	10	10	4	8	11	14	12	18	
*n* experts^b^			18	13	12	9	18	37	13	19	

a The Netherlands, Belgium, UK, Germany, Romania, Italy, Hungary and Spain.

b Some of the experts represented several profiles as per working in several setting.

Participants identified up to 31 documents regulating PS: Spain (*n* = 11),^5–15^ The Netherlands (*n* = 4),^16–19^ UK (*n* = 4),^2^^,^^20–23^ Italy (*n* = 4),^23–26^ Belgium (*n* = 3),^27–29^ Hungary (*n* = 2)^30^^,^^31^ and Germany (*n* = 2).^32^^,^^33^ Diverse regulations were reported: end-of-life laws (*n* = 12), professional guidelines (*n* = 10), general health care laws (*n* = 10) and palliative care laws (*n* = 2). The scope of these regulations applies to all health care professionals at a national level, except for Spain, where mainly regional norms apply for certain territories (table 4).

**Table 4:** Reported regulations, norms and professional directives

Country (*n*)	Title of regulation	Type	Year	Institution or organization	Scope of application
Belgium (3)	Loi relative aux soins palliatifs, 2002 (Palliative Care law, 2002)^27^	PC Law^a^	2002	Ministry of social affairs, public health and environment	National
	Law on Patient rights^28^	General law	2002	Ministry of social affairs, public health and environment	National
	Federatie palliatieve zorg—medische beslissingen levenseinde (Palliative care federation—end-of-life medical decisions)^29^	Guideline	2013	Flanders PC federation	Regional
Germany (2)	Civil Law Codex §§ 630a to 630 h BGB (Civil Law Codex) an the §§ 1901a to 1904 BGB^32^	General law	2002	Ministry of Justice and protection for the consumer	National
	German Criminal Code Book §§ 223 ff. StGB^33^	General law	1998	Ministry of Justice and protection for the consumer	National
Hungary (2)	1997. évi CLIV. törvény az egészségügyről (Health Care Law, CLIV)^30^	General law	1997	Ministry of Health	National
	Az Emberi Erőforrások Minisztériuma szakmai irányelve a daganatos felnőtt betegek teljes körű hospice és palliatív ellátásáról (Palliative and Hospice Care Clinical Practice Guidelines)^31^	Guideline	2017	Ministry of Human Resources	National
Italy (4)	Law on informed consent and advance treatment directives 219/2017^23^	EoL^b^	2017	President of the Council of Ministers	National
	Legge 38 del 2010 sulle cure palliative. Disposizioni per garantire l'accesso alle cure palliative e alla terapia del dolore^24^	PC Law^a^	2010	Ministry of Economics and finances	National
	Sedazione palliativa profonda continua nelĺimminenza della morte. Comitato nazionale bioetica 29/1/2016^25^	Guideline	2016	National Bioethics Committee	National
	Le Cure di fine vita e ĺanestesia rianimatore: Raccomdanzioni SIAARTI per ĺapproccio alla persona morente Update 2018^26^	Guideline	2018	Italian Society for Anesthesia, analgesia, reanimation and intensive therapy	National
The Netherlands (4)	Dutch Civil Code https://wetten.overheid.nl/BWBR0005290/2012-06-13#Boek7_Titeldeel7_Afdeling5^16^	General law	2012	Ministry of Justice and Safety	National
	Law for Medical Treatment Contracts (WGBO), https://wetten.overheid.nl/BWBR0007021/2006-02-0117	General law	2006	Ministry of Justice and Safety	National
	IKNL guidelines (www.pallialine.nl)^18^	Guideline	2009	Integraal Kancer Centrum Nederand, IKNL	National
	KNMG guideline on Palliative Sedation^19^	Guideline	2009	Dutch Medical Association KNMG	National
Spain (11)	Ley general de Sanidad, 1986^5^	General law	1986	Ministry of Health	National
	Law on Rights and Guarantees of Persons in the Process of Dying from Madrid Community (4/2017)^6^	EoL^b^	2017	Madrid Regional Government	Regional
	Ley de derechos y garantías de la dignidad de la persona en el proceso de la muerte, Andalusian Community (Ley 2/2010, de 8 de abril)^8^	EoL^b^	2010	Andalucía Regional Government	Regional
	Ley 5/2015, de 26 de junio, de derechos y garantías de la dignidad de las personas enfermas terminales^9^	EoL^b^	2015	Galicia Regional Government	Regional
	Ley de derechos y garantías de la dignidad de la persona en el proceso de morir y de la muerte Ley Aragón 2011^10^	EoL^b^	2011	Aragón Regional Government	Regional
	Ley de derechos y garantías de la dignidad de la persona en el proceso de la muerte, Navarra 2011^11^	EoL^b^	2011	Navarra Regional Government	Regional
	Ley de derechos y garantías de la dignidad de la persona en el proceso de la muerte, Canarias 2015^12^	EoL^b^	2015	Canarias Regional Government	Regional
	Ley 4/2015 de Derechos y Garantías de la persona en el proceso de morir^13^	EoL^b^	2015	Baleares Regional Government	Regional
	Ley 5/2018 sobre Derechos y Garantías de la dignidad de las personas en el proceso del final de la vida (Asturias)^14^	EoL^b^	2018	Asturias Regional Government	Regional
	Ley 11/2016 de garantía de los derechos y de la dignidad de las personas en el proceso final de su vida^15^	EoL^b^	2016	Euskadi Regional Government	Regional
	Ley 16/2018 de derechos y garantías de la dignidad de la persona en el proceso de atención al final de la vida^7^	EoL^b^	2018	Valencia Regional Government	Regional
UK (5)	National Palliative Care guideline	Guideline	2016	National Institute for Health and Care Excellence	/
	Treatment and care towards the end of life: good practice in decision-making^21^	Guideline		General Medical council* (statutory regulator for the medical profession in UK)	National
	Palliative Care Formulary version 6^22^	Guideline	2018	/	/
	EAPC Framework for Palliative Sedation^2^	Guideline	2009	European Association for Palliative Care	/
	Mental Capacity Act 2005 and related code of conduct^20^	General law	2005		

a Palliative care law.

b End-of-life regulation.

Across countries, PS is normally understood as a common medical practice and, in this sense, the consulted professionals understand that it is affected by the general law that regulates medical practice without explicit references to PS. In Spain, in 12 regional (autonomous) laws, PS is explicitly recognized as a right of the patient at the end of life when there are refractory symptoms.^5–15^ In Italy, the law of informed consent and advance directives specifically recognizes the doctor can use deep sedation when there are refractory symptoms.^23^ There are also laws that do not directly refer to the sedation practice but are relevant to PS as they regulate related principles included in the EAPC Framework for PS: the obligation of doctors to respect advance directives (all countries), the informed consent of the patient (Belgium, Germany, Hungary, Italy, the Netherlands and Spain), issues around the decision-making process (Belgium, Hungary, Italy, Netherlands, Spain and the UK) and the obligation to document the whole treatment process (Belgium, Germany, Italy, the Netherlands and the UK). Furthermore, in countries like Germany, the Netherlands and the UK, PS professional guidelines possess normative significance and regulate PS via professional bodies. They are binding as good practice in terms of disciplinary law (Supplementary Annex 2).

### Belgium

In Belgium, the law on patients’ rights intended to help or accompany a dying patient (art. 2), applies to any medical care provided by a professional to a patient (art. 3) without mentioning PS. It covers some PS-related EAPC principles: the duty to respect patients’ right (art. 5–7), the informed consent (art. 8), the obligation to honour the patient’s advance directives (art. 8.5) and the documentation of the entire decision process in medical files (art. 9).^28^ The palliative care law requires the informed consent ‘for all evaluations and treatments’ (art. 7).^27^ In the Flanders region, a PS guideline was reported.^29^ Some features regarding sedation included: informed consent, unbearable suffering due to one or more refractory symptoms (physical, and/or psychological end/or existential), discussion of nutrition and hydration, and the principle of proportionality.

Generally, the purpose of PS is symptom control and the alleviation of the patient’s suffering must be regarded as a medical action within the meaning of the Health Care Professions Exercise Act (art. 2). Proportional sedation is started in consensus with the patient (or their representative in case of limited decision-making capacities), for refractory symptoms causing unbearable suffering. It is accepted that the task of doctor extends beyond strictly curative and preventive treatments.^34^ There is a social consensus that a physician must also assist the patient at the end of life and adequately alleviate the patient’s suffering and pain [art 2^27^, art 8 and 11bis^28^]. This medical action is permitted to the extent that it is carried out with due care and diligence. PS can only take place when the indication and application conditions are met (e.g. terminal prognosis, consent, intent to symptom control, proportionality).

### Germany

The German health care system relies on self-regulation between health care providers and sickness funds, and government regulations are kept as limited as possible. In accordance, medical practice is regulated by recommendations produced by medical associations (adapted from the EAPC Framework),^2^ and there are only few regulations in the German penal or civil law. It is the regular body of medical law that applies to PS without explicit reference. The German Civil Code includes EAPC PS-related topics such as patients’ consent (630d), the obligation for providing information (630e) and the documentation of the entire decision-making process in medical files (630f).^32^ It determines the obligation of doctors to honour advance directives (section 1901a and b on ‘living wills’).^32^

In general, laws and jurisdiction allow PS as long as the treatment goal definitely is symptom relief and does not intend to hasten dying. There is a clear understanding that giving medications for the relief of suffering at the end of life with the risk of shortening life is permissible under the penal code, because this does not count as bodily harm when applied with the consent of the patient or legal representative, and according to state-of-the-art. In comparison, articles 223 and 224 from the German Criminal Code regulate bodily harm and dangerous bodily harm, respectively; regarding the punishment of ‘whoever causes harm by administering poison or other substances which are harmful to health (…)’.^33^

### Hungary

The Hungarian general health law is the essential regulation for health care and is relevant to PS. It defines patients’ rights including the right to self-determination (informed consent) and the right to refuse health care or certain medical treatments. Life-supporting and life-saving interventions may only be refused if the patient suffers from a serious incurable illness that would lead to death within a short time period. A person is also allowed to make an advance directive to refuse medical treatments (including life-supporting and life-saving interventions) and/or name a durable power of attorney for his future incapacity. The duty to respect the patients’ right, and the obligation of doctors to honour advance directives are also included.^30^

PS is regulated in more detail by a clinical guideline issued in a ministerial decree.^31^ The term used in the guideline is terminal PS that aims to reduce intolerable and refractory symptoms (listed in text). Two forms of PS are distinguished in this regulation: the short-term, ‘mild’ or intermittent, and the continuous, deep sedation. In the case of a non-critical situation, the applicability of PS should be based on the patient’s advance directives. Before starting sedation, the patient’s relative or durable power of attorney must be informed about the procedure, its benefits and potential risks. Ethical considerations, indications, medication and practice of artificial hydration are also described.

### Italy

In Italy, there is a legal framework regarding sedation in the form of an end-of-life regulation on informed consent containing EAPC PS-related topics such as refractory symptoms (art 2), informed consent (art. 1, 2), the duty to respect patients’ rights (art. 2) and the obligation to document the sedation process (art. 2).^23^ The palliative care law itself does not explicitly refer to PS although documentation of the entire decision-making process in medical files maybe inferred (art. 7).^24^

Furthermore, professional guidelines explicitly regulating PS practice are available. The National Bioethics Committee includes—besides a chapter about patients’ informed consent—the norms regarding palliative care applying to PS as well (p16).^25^ Some of these issues are the availability of opioids,^35^ empowering analgesic therapies,^36^ promotion of quality of life of patient specially in terminal stages, the specific reference to advanced and terminal stages (art. 5, c. 3),^24^ the 2007 recommendations on PS of the Italian Society for Palliative Care and the Code on Medical Deontology 2014 that mandates doctors to alleviate suffering until death (art. 3,39). The SIAARTI guideline mentions regulations regarding the informed consent and advanced directives: Law 219/17 particularly referred to the consensus on advanced care planning (art. 5).^23^^,^^26^

### The Netherlands

In the Netherlands, PS is considered a normal medical practice. By implication, the Dutch Medical Association (KNMG) guideline for PS is part of the physician’s professional standard(s).^19^ By law, the doctors (and nurses) are obliged to act according to professional standard(s) as for its legal status and significance. The government urged the medical profession to draft a national guideline on terminal sedation around 2005, after which the KNMG agreed to appoint a multidisciplinary committee accordingly. The guidelines were adopted by the KNMG’s executive committee in 2005, and presented to the House of Representatives of the States-General. The guideline describes the conditions under which PS is considered good medical practice.^19^ Being part of the professional standard, they also possess legal significance. In January 2006, the Public Prosecution Service stated that it saw no reason to prosecute doctors who adhere to the guideline. Any doctor who deviates from them must bear in mind that provides inaccurate medical care.^19^ Besides, the *Integral Kancercentrum Nederland* guideline of 2005 was updated in 2009 and is currently updated again.^18^

The Dutch Civil Code regulates the agreement of medical treatment (Book 7, title 7, section 5).^17^ It is a mandatory law applying to any medical treatment agreement between patient and any health care provider. It regulates widely EAPC PS-related principles without mentioning PS: the informed consent, the duty to respect the patient’s right, the transparency of the decision-making process, the collegial decision-making, the documentation of the entire decision-making process and the obligation to honour advance directives (art 450).^16^

### Romania

Currently in Romania, there exists the general law 95/2006 regarding health care, but it does not specifically refer to palliative care services.^37^ The patients’ rights law 46/2003 includes the right to informed consent and the right to refuse health care or certain medical treatments. Guidelines established by the palliative care protocols are used only locally in palliative care services.^38^ To date, no discussions about legal aspects of PS are in place.

### Spain

PS is explicitly regulated through regional end-of-life regulations on rights and guarantees of persons in the process of dying, all of which have an article on the persons’ right to receive PS when there are refractory symptoms.^5–15^ EAPC PS-related principles such as the informed consent and the obligations of clinical staff of informing and respecting advance directives are generally observed. Normally, this legal corpus approaches a definition for PS as well as for refractory symptoms, and the recognition of the patient’s right to PS if there are refractory symptoms. Sometimes this is recognized under an article on ‘the patient’s right to receive integral palliative care and pain treatment’, while sometimes there is a specific article stating the ‘patient’s right to the administration of palliative sedation’. Besides, the law 41/2002 on Patients Autonomy (art. 9.3a.) refers to the adequate consent for PS, and the General Health Law 1986 mentions PS specifically.^5^

### The UK

There is no formal legal framework specifically addressing PS (mostly because this is used differently in the UK), but there are legal requirements relevant to PS around advance directives and mental capacity (points 24–26) and requirements around consent and care in the law in general.^20^ The General Medical Council produced ethical guidance on the ‘Treatment and care towards the end of life’.^21^ It acts as the statutory regulator for the medical profession and is consistent with current laws on decision-making for patients who lack capacity^20^^,^^39^; the law prohibiting killing (including euthanasia) and assisting suicide; and the requirements of the Human Rights Act 1998. However, it is not intended as a statement of the legal principles or a substitute for legal advice. Ethical guidance from the General Medical Council includes EAPC PS-related topics such as decision capacity and making decisions (14c), collegial decision-making (16e), meeting a patients’ nutrition and hydration needs, the role of relatives, documentation of the entire decision-making process in medical files (rec. 61). Other topics covered are advance care planning (recs. 63–67).^21^

There is also case law; a number of cases relate to use of medication at end of life—whether for symptom control or euthanasia. Amongst the key points, some of the topics include the duty to respect the patient’s right and the consent.

There are two clinical guidelines regulating PS practice: NICE Guideline: End of life care for adults: service delivery; and the NICE Guideline Care of dying adults in the last days of life, which—while not law—are expected to be part of best practice, and might be considered part of legal proceedings. Also the Palliative Care Formulary version 6^22^ and the EAPC Framework on Palliative Sedation^2^ are observed.

## Discussion

There are broadly two diverse regulatory approaches to PS: one regulating medical care at a general level (e.g. requiring informed consent) and another regulating particular PS procedures. Most countries do regulate PS indirectly through general laws that concern normal medical practice and contain relevant elements to PS according to the EAPC PS Framework such as the general obligation of doctors to honour advance directives, the informed consent of the patient, etc. In the Netherlands, the UK and Germany, PS is regulated through PS professional guidelines. They have normative significance and professionally regulate PS. Some countries’ laws do include specific recognition of the right to receive PS. In Spain, PS is recognized as a patient’s right through End-of-life laws when there are refractory symptoms^5–15^; and in Italy, the law on informed consent and advance treatment directives acknowledges that the doctor may use profound sedation in the same circumstances.^23^ Other countries like France have regulated PS through the Claeys–Leonetti Law 2016-87, recognizing the right to ‘a deep and continuous sedation causing an alteration of consciousness maintained until death’.

The majority of identified documents do not regulate the intention or aim of the practitioner’s intervention. Only in laws and professional guidelines that explicitly regulate PS, it is stated that the aim of the intervention is to palliate suffering. Medical interventions are dependent on many variables and always have a risk of adverse outcomes. For this reason, medical laws may regulate the means, the structure and processes such as use of medicines to reduce consciousness, informed consent, etc. PS is normally understood as a medical procedure and, therefore, applicable regulations are similar to other medical acts.

This exploratory fieldwork study has enabled an understanding of how PS is regulated. It has retrieved a rich harvest of regulations, but in turn, it needs to acknowledge limitations. Even if the study was not aimed at collecting representative opinions from each discipline and country but, rather, to identify and comparatively analyze relevant regulations, the number of participants per country does not correspond to the number of inhabitants. Also, it is based on a sample of physicians predominantly working in palliative care contexts. This may be explained because PS means different things in different countries or may be little used.^40^ In most countries, the term ‘palliative sedation’ seems not much used outside the field of palliative care and practices in other fields might be different. Likewise, another limitation is the diversity of environments in which palliative care is delivered and the variability of models between countries. Furthermore, given that PS can be understood as continuous or intermittent, as deep or light; it is possible that identified regulations also apply broadly to the various forms of sedation.

In Health Governance, understood as the total set of regulations in place, looking for good practices is key to find health references and may, in turn, be decisive for adequate policy practices. Examples in countries point out some regulated topics that may have an impact on end-of-life care: informed consent, refractory symptoms descriptions, etc.; but overall, shows how some countries rely on the evidence-based professional guidelines for an optimal sedation practice. Future research could define if regulations are indeed favouring or hindering optimal PS, and therefore, estimating the impact of regulations on patients’ wellbeing. A possible approach could be the analysis of the correlation between the two main regulatory approaches with clinical outcomes, addressing professionals’ routines regarding PS.

## Supplementary data

Supplementary data are available at *EURPUB* online.

## Supplementary Material

ckac153_Supplementary_Data
