# Supplementary material for: Regulations on palliative sedation: an international survey across eight European countries
Source: Eur J Public Health. 2022 Oct 27;33(1):35–41. doi: 10.1093/eurpub/ckac153 (PMC9897985; doi:10.1093/eurpub/ckac153)
Supplement: ckac153_Supplementary_Data [file ckac153_supplementary_data.zip › ckac153_Supplementary_Data/ejph-2021-11-om-1074-File002.pdf]

Palliative Sedation

The project

Dear Expert

We are inviting you to take part in this survey as an expert in Palliative Sedation related issues in your country.

This survey aims to identify regulations affecting palliative sedation, **in healthcare practice in Belgium, Germany, Hungary, Italy, the Netherlands, Romania, Spain, and the United Kingdom. It is part of a larger EU Horizon2020 Research Project called '**<u>**Palliative Sedation' – The use of proportional palliative sedation for the relief of refractory symptoms**</u>**".**

This survey will take you about 5 minutes to complete.

This study has been approved by the Ethical Committee of the University of Navarra registered under number 2019.194. Your participation in this survey is voluntary. Because we are not including identifiers in the final database, there will be no way to connect your details to your answers other than through a password-protected document available only to the Spanish partner´s members. We believe there is minimal risk in undertaking this survey. Once all data have been analysed, your original questionnaire will be destroyed. If you wish your name and organisation to be made public and acknowledged in the final report and paper, there will be an option allowing you to do so below.

You may withdraw from the study at any time by contacting Eduardo Garralda (egarralda@unav.es). You have the right to access your personal data, modify them, cancel them or decline public acknowledgement if you wish, as well as to exercise other rights that are listed on the <u>**Data Protection Page**</u>**, where you can obtain more details about the University of Navarra privacy policy. If you wish to clarify anything or exercise your rights, please contact** <u>**dpo@unav.es**</u>**.**

Information from the survey will be used to prepare a scientific paper and report. It will also be used to inform a revision to the EAPC Framework on Palliative Sedation. We would be very grateful if you could complete this brief survey. By continuing with the survey, you agree to take part in this study.

We thank you for your interest and participation in this study.

Yours sincerely,

Eduardo Garralda
Research Assistant,
Institute for Culture and Society, University of Navarra.
Email: egarralda@unav.es

This survey has been designed by the ATLANTES Research Team, at the Univeristy of Navarra (Spain), in collaboration with all project partners:

Radboud University MC, Nijmegen, The Netherlands
University of Pécs, Hungary
Katholieke Universiteit Leuven, Belgium
Hospice Casa Sperantei, Romania
European Association for Palliative Care
Lancaster University, United Kingdom
La Maddalena S.P.A, Italy
Universitaet Klinikum Bonn, Germany

The survey

The questions were carefully chosen following a review of previous national and international surveys and questionnaires about Palliative Sedation. We selected questions after review by the research team and discussion with all project partners.

The survey consists of 5 questions. You can exit the survey at any time. Please answer the questions to the best of your ability. Some of the questions may require you to give your best estimate as an expert in Palliative Sedation issues in your country.

We have provided definitions before each section as well as additional information at the end of the section in order to assist you. Please read the definitions before starting to answer the questions. If you have any comments that you wish to add, there is a comment box at the end of the whole survey.

You can also download the questionnaire in a pdf format, so that you can read the questions before you complete it or by using the pdf form and returning it to us via e-mail if you prefer.

* 1. **Contact Information**

Name (as you would like it to appear in the Acknowledgements) ____________________

Affiliation (as you would like it to appear in the Acknowledgements) ____________________

Country ____________

2. **You will not be formally acknowledged in the acknowledgement section of the final report. If you wish to be publicly acknowledged please tick the box below.**

☐ I would like to publicly acknowledged.

Regulations affecting Palliative Sedation

Definitions and relevant information

We use the following definition for regulations: regulations are categories of delegated decision-making involving the use of secondary legislation; various types for the implementation of socioeconomic policy objectives and includes laws. Laws are rules that govern behavior. Laws can be made by a legislature, resulting in primary legislation (often called statutes or acts), by executive or local government through the issue of secondary legislation (including decrees, regulations and bylaws), or by judges through the making of binding legal precedent (normally in common law jurisdictions) (Clarke, 2016, p. vii)

3. **Is there a legal framework for Palliative Sedation in your country?**

- ○ No
- ○ Yes
- ○ I do not know

Comments

4. **If yes, which of the following regulations exist in your country with regard to Palliative Sedation?**

- ☐ General Health law
- ☐ Specific law on Palliative Sedation
- ☐ Palliative Care law that refers to Palliative Sedation
- ☐ Decrees relating to certain features of Palliative Sedation
- ☐ End of life regulations that mention Palliative Sedation
- ☐ Others
- ☐ None
- ☐ If others, please specify

5. **If you have ticked any of the above, could you please provide the name of the regulation and a weblink?**

6. **At what level does this regulation apply in your country?**

	Level of application
General Health law	
Specific law on Palliative Sedation	
Palliative Care law that refers to Palliative Sedation	
Decrees relating to certain features of Palliative Sedation	
End of life regulations that mention Palliative Sedation	
Others	

If others, please specify

<u>For these questions we have used the following sources:</u>

-Arias-casais, N. Consensus Building on Health Indicators to Assess PC Global Development With an International Group of Experts. Journal of Pain and Sym Manag, 2019.

-Khader MM. The Use of Palliative Sedation for Terminally Ill Patients: Review of the Literature and an Argumentative Essay. J Palliat Care Med. 2015;5(4).

Palliative Sedation

Thank you very much for your time!

This is the end of the survey

7. Please feel free to make any other comments here
