# Supplementary material for: Regulations on palliative sedation: an international survey across eight European countries
Source: Eur J Public Health. 2022 Oct 27;33(1):35–41. doi: 10.1093/eurpub/ckac153 (PMC9897985; doi:10.1093/eurpub/ckac153)
Supplement: ckac153_Supplementary_Data [file ckac153_supplementary_data.zip › ckac153_Supplementary_Data/ejph-2021-11-om-1074-File003.docx]

#### Annex 2. Reference to palliative sedation and adherence to the principles of the EAPC framework on Palliative Sedation across regulations

| **Country*** | **Regulation´s title** | **Mention Palliative Sedation/**  (x/√) | **Topics covered (based on the EAPC Framework on Palliative Sedation)** | | | | | | | | | | | |
| --- | --- | --- | --- | --- | --- | --- | --- | --- | --- | --- | --- | --- | --- | --- |
| (n) |  |  | Refractory Symptom | Patient type | Informed consent | Procedure Requirements | Nutrition & hydration | Patients right | Decision making | Collegial decision-making | Info. relatives | Document. process | Proportionality | Obligation to honor advance directives |
| **Belgium**  (3) | [Loi relative aux soins palliatifs, 2002](http://files.palliatieve.org/download/76/Wet_140602_pall_zorg.pdf)) | X |  |  |  |  |  |  |  |  |  |  |  |  |
|  | [Law on Patient rights](http://www.ejustice.just.fgov.be/cgi_loi/loi_a1.pl?language=nl&la=N&cn=2002082245&table_name=wet&&caller=list&fromtab=wet&tri=dd+AS+RANK) | X | **√** |  | **√** |  |  | **√** | **√** |  |  | **√** | **√** | **√** |
|  | [Federatie palliatieve zorg - medische beslissingen levenseinde](http://www.palliatief.be/accounts/143/attachments/Brochures/brochure_med_begel_sterven_v2_13_12_2013.pdf) (Palliative care federation - end-of-life medical decisions) | X | **√** |  | **√** |  | **√** |  |  |  |  |  | **√** |  |
| **Germany**  (2) | [§§ 630a to 630 h BGB (Civil Law Codex) an the §§ 1901a to 1904 BGB](http://www.gesetze-im-internet.de/englisch_bgb/englisch_bgb.html#p2646) | X |  |  | **√** |  |  |  |  |  | **√** | **√** |  | **√** |
|  | German Criminal Code Book [§§ 223 ff. StGB](https://www.gesetze-im-internet.de/englisch_stgb/englisch_stgb.html) | X |  |  |  |  |  |  |  |  |  |  |  |  |
| **Hungary**  (2) | Health Care law, CLIV <https://eletveg.hu/hu/egeszsegugyi-kerdesek/> | X |  |  | **√** |  |  | **√** |  |  |  |  |  | **√** |
|  | Professional directives regarding Palliative and hospice care [Az Emberi Erőforrások Minisztériuma szakmai irányelve a daganatos felnőtt betegek teljes körű hospice és palliatív ellátás](http://www.hbcs.hu/uploads/jogszabaly/2578/fajlok/EEMI_szakmai_iranyelve_daganatos.pdf) | **√** | **√** |  | **√** |  | **√** |  | **√** |  | **√** |  | **√** | **√** |
| **Italy**  (4) | [Law on informed consent and advance treatment directives 219/2017](http://www.salute.gov.it/portale/dat/dettaglioContenutiDat.jsp?lingua=italiano&id=4953&area=dat&menu=vuoto) | **√** | **√** |  | **√** |  |  | **√** |  |  |  | **√** |  |  |
|  | Law on Palliative Care [Legge 38 del 2010 sulle cure palliative. Disposizioni per garantire l'accesso alle cure palliative e alla terapia del dolore](http://www.trovanorme.salute.gov.it/norme/dettaglioAtto?aggiornamenti=&attoCompleto=si&id=32922&page=&anno=null) | X |  |  |  |  |  |  | **√** |  |  | **√** |  |  |
|  | [Sedazione palliativa profonda continua nell´imminenza della norte, 29/1/2016](http://bioetica.governo.it/media/1804/p122_2016_sedazione_profonda_it.pdf) | **√** | **√** |  | **√** |  |  | **√** |  | **√** |  |  |  | **√** |
|  | [Le cure di fine vita e l´anestesia rianimatore: Raccomandazioni SIAARTI per l´ approcio alla persona morente. Update 2018](http://www.siaarti.it/Ricerca/documento-siaarti-fine-vita.aspx) | X |  |  | **√** |  |  |  |  |  |  |  |  | **√** |
| **Netherla-nds**  (4) | Dutch Civil Code [https://wetten.overheid.nl/BWBR0005290/2012-06-13#Boek7_Titeldeel7_Afdeling5](https://wetten.overheid.nl/BWBR0005290/2012-06-13%23Boek7_Titeldeel7_Afdeling5%20%20) | X |  |  | **√** |  |  | **√** | **√** | **√** |  | **√** |  | **√** |
|  | Law for Medical Treatment Contracts (WGBO), <https://wetten.overheid.nl/BWBR0007021/2006-02-01> | X |  |  |  |  |  |  |  |  |  |  |  |  |
|  | IKNL guidelines ([www.pallialine.nl](file:///C:\Users\bucaaa.b.jpte\Downloads\www.pallialine.nl)) | **√** |  |  |  |  |  |  |  |  |  |  |  |  |
|  | [KNMG guideline on Palliative Sedation](https://www.knmg.nl/advies-richtlijnen/knmg-publicaties/publications-in-english.htm) | **√** | **√** | **√** |  |  |  |  | **√** |  | **√** |  | **√** |  |
| **Spain**  (11) | [Ley general de Sanidad, 1986](https://www.eutanasia.cat/attachments/article/11/llei_general_de_sanitat_14-1986.pdf) | **√** |  |  |  |  |  |  |  |  |  |  |  |  |
|  | [Law on Rights and Guarantees of Persons in the Process of Dying from Madrid Community (4/2017)](http://www.madrid.org/wleg_pub/secure/normativas/contenidoNormativa.jsf?opcion=VerHtml&nmnorma=9690&cdestado=P#no-back-button) | **√** | **√** | **√** | **√** |  |  | **√** | **√** |  | **√** |  |  | **√** |
|  | [Ley de derechos y garantías de la dignidad de la persona en el proceso de la muerte, Andalusian Community (Ley 2/2010, de 8 de abril9](https://www.juntadeandalucia.es/boja/2010/88/1) | **√** | **√** |  | **√** |  |  | **√** |  |  |  |  |  | **√** |
|  | [Ley 5/2015, de 26 de junio, de derechos y garantías de la dignidad de las personas enfermas terminales](https://www.boe.es/buscar/pdf/2015/BOE-A-2015-10200-consolidado.pdf) | **√** | **√** |  |  |  |  | **√** |  |  |  |  |  |  |
|  | [Ley de derechos y garantías de la dignidad de la persona en el proceso de morir y de la muerte Ley Aragón 2011](https://www.boe.es/boe/dias/2011/05/14/pdfs/BOE-A-2011-8403.pdf) | **√** | **√** |  |  |  |  | **√** |  |  |  |  |  | **√** |
|  | [Ley de derechos y garantías de la dignidad de la persona en el proceso de la muerte, Navarra 2011](https://www.boe.es/buscar/pdf/2011/BOE-A-2011-7408-consolidado.pdf) | **√** | **√** |  | **√** |  |  | **√** |  |  |  |  |  | **√** |
|  | [Ley de derechos y garantías de la dignidad de la persona en el proceso de la muerte, Canarias 2015](https://www.boe.es/boe/dias/2015/03/04/pdfs/BOE-A-2015-2295.pdf) | **√** | **√** |  | **√** |  |  | **√** |  |  |  |  |  | **√** |
|  | [Ley 4/2015 de Derechos y Garantías de la persona en el proceso de morir](https://www.boe.es/buscar/pdf/2015/BOE-A-2015-4332-consolidado.pdf) | **√** | **√** |  | **√** |  |  | **√** |  |  |  |  |  | **√** |
|  | [Ley 5/2018 sobre Derechos y Garantías de la dignidad de las personas en el proceso del final de la vida (Asturias)](https://www.boe.es/boe/dias/2018/07/27/pdfs/BOE-A-2018-10580.pdf) | **√** | **√** |  | **√** |  |  | **√** |  |  |  |  |  |  |
|  | [Ley 11/2016 de garantía de los derechos y de la dignidad de las personas en el proceso final de su vida](https://www.boe.es/boe/dias/2016/07/21/pdfs/BOE-A-2016-6997.pdf) | **√** | **√** |  | **√** |  |  | **√** |  |  |  |  |  |  |
|  | [Ley 16/2018 de derechos y garantías de la dignidad de la persona en el proceso de atención al final de la vida](https://www.boe.es/buscar/pdf/2018/BOE-A-2018-10760-consolidado.pdf) | **√** | **√** |  | **√** | **√** |  | **√** | **√** |  | **√** |  |  | **√** |
| **United Kingdom**  (5) | [Mental Capacity Act 2005 and related code of conduct](http://www.legislation.gov.uk/ukpga/2005/9/contents) | X |  |  |  |  |  |  |  |  |  |  |  | **√** |
|  | [National Palliative Care guideline](http://www.hbcs.hu/uploads/jogszabaly/2578/fajlok/EEMI_szakmai_iranyelve_daganatos.pdf) | X |  |  |  |  |  |  |  |  |  |  |  |  |
|  | [Treatment and care towards the end of life: good practice in decision making](https://www.gmc-uk.org/ethical-guidance/ethical-guidance-for-doctors/treatment-and-care-towards-the-end-of-life) (decision-making, advanced care directives, nutrition and hydration) | X |  |  |  |  | **√** |  | **√** | **√** | **√** | **√** |  | **√** |
|  | [Palliative Care Formulary version 6](https://www.pharmpress.com/product/9780857113481/palliative-care-formulary-pcf6) | X |  |  |  |  |  |  |  |  |  |  |  |  |
|  | [EAPC Guidelines on Palliative Sedation](https://www.eapcnet.eu/home/ArtMID/1395/ArticleID/735/eapc-recommended-framework-for-the-use-of-sedation-in-palliative-care) | **√** |  |  |  |  |  |  |  |  |  |  |  |  |

*Romanian participants did not report any regulation applying to palliative sedation.
